# Supplementary material for: Unveiling the Bioactive Potential of the Invasive Jellyfish Phyllorhiza punctata Through Integrative Transcriptomic and Proteomic Analyses
Source: Biomolecules. 2025 Aug 4;15(8):1121. doi: 10.3390/biom15081121 (PMC12383608; doi:10.3390/biom15081121)
Supplement: Supplementary file 1 [file biomolecules-15-01121-s001.zip › Suplementary Material/FileS3/PEPFOLD4/XP_065052612_6303/clusters.html]

 PEP-FOLD clusters


Download archive of all models  here   

|  |  |  |
| --- | --- | --- |
| # | file | sOPEP |
| # cluster 1 - Size: 9 |  |  |
|  | XP\_065052612\_6303-model1 | -22.8216 |
|  | XP\_065052612\_6303-model1.1 | -22.6337 |
|  | XP\_065052612\_6303-model1.2 | -22.5704 |
|  | XP\_065052612\_6303-model1.3 | -22.5704 |
|  | XP\_065052612\_6303-model1.4 | -22.553 |
|  | XP\_065052612\_6303-model1.5 | -22.4243 |
|  | XP\_065052612\_6303-model1.6 | -22.2642 |
|  | XP\_065052612\_6303-model1.7 | -21.5874 |
|  | XP\_065052612\_6303-model1.8 | -18.9387 |
| # cluster 2 - Size: 4 |  |  |
|  | XP\_065052612\_6303-model2 | -18.8934 |
|  | XP\_065052612\_6303-model2.1 | -18.4814 |
|  | XP\_065052612\_6303-model2.2 | -18.4285 |
|  | XP\_065052612\_6303-model2.3 | -18.1779 |
| # cluster 3 - Size: 4 |  |  |
|  | XP\_065052612\_6303-model3 | -18.429 |
|  | XP\_065052612\_6303-model3.1 | -18.1466 |
|  | XP\_065052612\_6303-model3.2 | -18.0993 |
|  | XP\_065052612\_6303-model3.3 | -16.6675 |
| # cluster 4 - Size: 3 |  |  |
|  | XP\_065052612\_6303-model4 | -18.1704 |
|  | XP\_065052612\_6303-model4.1 | -17.0371 |
|  | XP\_065052612\_6303-model4.2 | -16.6784 |
| # cluster 5 - Size: 1 |  |  |
|  | XP\_065052612\_6303-model5 | -17.0811 |
| # cluster 6 - Size: 13 |  |  |
|  | XP\_065052612\_6303-model6 | -16.6458 |
|  | XP\_065052612\_6303-model6.1 | -16.5217 |
|  | XP\_065052612\_6303-model6.2 | -16.5132 |
|  | XP\_065052612\_6303-model6.3 | -16.4938 |
|  | XP\_065052612\_6303-model6.4 | -16.4213 |
|  | XP\_065052612\_6303-model6.5 | -16.4094 |
|  | XP\_065052612\_6303-model6.6 | -16.3007 |
|  | XP\_065052612\_6303-model6.7 | -16.2598 |
|  | XP\_065052612\_6303-model6.8 | -16.2545 |
|  | XP\_065052612\_6303-model6.9 | -16.0089 |
|  | XP\_065052612\_6303-model6.10 | -15.9838 |
|  | XP\_065052612\_6303-model6.11 | -15.9262 |
|  | XP\_065052612\_6303-model6.12 | -15.5417 |
| # cluster 7 - Size: 4 |  |  |
|  | XP\_065052612\_6303-model7 | -16.369 |
|  | XP\_065052612\_6303-model7.1 | -16.3335 |
|  | XP\_065052612\_6303-model7.2 | -16.196 |
|  | XP\_065052612\_6303-model7.3 | -16.1865 |
| # cluster 8 - Size: 1 |  |  |
|  | XP\_065052612\_6303-model8 | -16.2777 |
| # cluster 9 - Size: 3 |  |  |
|  | XP\_065052612\_6303-model9 | -16.1418 |
|  | XP\_065052612\_6303-model9.1 | -16.1025 |
|  | XP\_065052612\_6303-model9.2 | -15.8913 |
| # cluster 10 - Size: 3 |  |  |
|  | XP\_065052612\_6303-model10 | -16.0881 |
|  | XP\_065052612\_6303-model10.1 | -15.9114 |
|  | XP\_065052612\_6303-model10.2 | -15.3898 |
| # cluster 11 - Size: 1 |  |  |
|  | XP\_065052612\_6303-model11 | -14.8888 |
| # cluster 12 - Size: 2 |  |  |
|  | XP\_065052612\_6303-model12 | -14.5305 |
|  | XP\_065052612\_6303-model12.1 | -14.2503 |
| # cluster 13 - Size: 2 |  |  |
|  | XP\_065052612\_6303-model13 | -13.8106 |
|  | XP\_065052612\_6303-model13.1 | -9.39144 |
| # cluster 14 - Size: 1 |  |  |
|  | XP\_065052612\_6303-model14 | -11.6529 |
| # cluster 15 - Size: 1 |  |  |
|  | XP\_065052612\_6303-model15 | -9.9885 |
| # cluster 16 - Size: 4 |  |  |
|  | XP\_065052612\_6303-model16 | -9.64836 |
|  | XP\_065052612\_6303-model16.1 | -8.78165 |
|  | XP\_065052612\_6303-model16.2 | -7.11589 |
|  | XP\_065052612\_6303-model16.3 | -7.07455 |
| # cluster 17 - Size: 1 |  |  |
|  | XP\_065052612\_6303-model17 | -9.34752 |
| # cluster 18 - Size: 2 |  |  |
|  | XP\_065052612\_6303-model18 | -9.26389 |
|  | XP\_065052612\_6303-model18.1 | -8.69943 |
| # cluster 19 - Size: 4 |  |  |
|  | XP\_065052612\_6303-model19 | -9.11585 |
|  | XP\_065052612\_6303-model19.1 | -7.87262 |
|  | XP\_065052612\_6303-model19.2 | -7.46965 |
|  | XP\_065052612\_6303-model19.3 | -7.45572 |
| # cluster 20 - Size: 4 |  |  |
|  | XP\_065052612\_6303-model20 | -8.7954 |
|  | XP\_065052612\_6303-model20.1 | -7.72806 |
|  | XP\_065052612\_6303-model20.2 | -7.0046 |
|  | XP\_065052612\_6303-model20.3 | -6.9975 |
| # cluster 21 - Size: 3 |  |  |
|  | XP\_065052612\_6303-model21 | -8.51918 |
|  | XP\_065052612\_6303-model21.1 | -7.12722 |
|  | XP\_065052612\_6303-model21.2 | -7.00303 |
| # cluster 22 - Size: 1 |  |  |
|  | XP\_065052612\_6303-model22 | -7.95826 |
| # cluster 23 - Size: 1 |  |  |
|  | XP\_065052612\_6303-model23 | -7.76411 |
| # cluster 24 - Size: 1 |  |  |
|  | XP\_065052612\_6303-model24 | -7.51023 |
| # cluster 25 - Size: 1 |  |  |
|  | XP\_065052612\_6303-model25 | -7.50047 |
| # cluster 26 - Size: 2 |  |  |
|  | XP\_065052612\_6303-model26 | -7.48049 |
|  | XP\_065052612\_6303-model26.1 | -7.37843 |
| # cluster 27 - Size: 2 |  |  |
|  | XP\_065052612\_6303-model27 | -7.43779 |
|  | XP\_065052612\_6303-model27.1 | -7.13732 |
| # cluster 28 - Size: 2 |  |  |
|  | XP\_065052612\_6303-model28 | -7.35207 |
|  | XP\_065052612\_6303-model28.1 | -6.92331 |
| # cluster 29 - Size: 1 |  |  |
|  | XP\_065052612\_6303-model29 | -7.26622 |
| # cluster 30 - Size: 1 |  |  |
|  | XP\_065052612\_6303-model30 | -7.23428 |
| # cluster 31 - Size: 1 |  |  |
|  | XP\_065052612\_6303-model31 | -7.01507 |
| # cluster 32 - Size: 1 |  |  |
|  | XP\_065052612\_6303-model32 | -6.96703 |
| # cluster 33 - Size: 2 |  |  |
|  | XP\_065052612\_6303-model33 | -6.96241 |
|  | XP\_065052612\_6303-model33.1 | -6.71475 |
| # cluster 34 - Size: 2 |  |  |
|  | XP\_065052612\_6303-model34 | -6.83117 |
|  | XP\_065052612\_6303-model34.1 | -6.6548 |
| # cluster 35 - Size: 1 |  |  |
|  | XP\_065052612\_6303-model35 | -6.82554 |
| # cluster 36 - Size: 1 |  |  |
|  | XP\_065052612\_6303-model36 | -6.82383 |
| # cluster 37 - Size: 1 |  |  |
|  | XP\_065052612\_6303-model37 | -6.78333 |
| # cluster 38 - Size: 2 |  |  |
|  | XP\_065052612\_6303-model38 | -6.76186 |
|  | XP\_065052612\_6303-model38.1 | -6.60494 |
| # cluster 39 - Size: 1 |  |  |
|  | XP\_065052612\_6303-model39 | -6.75615 |
| # cluster 40 - Size: 2 |  |  |
|  | XP\_065052612\_6303-model40 | -6.69751 |
|  | XP\_065052612\_6303-model40.1 | -6.56978 |
| # cluster 41 - Size: 1 |  |  |
|  | XP\_065052612\_6303-model41 | -6.61814 |
| # cluster 42 - Size: 1 |  |  |
|  | XP\_065052612\_6303-model42 | -6.46856 |
| # cluster 43 - Size: 1 |  |  |
|  | XP\_065052612\_6303-model43 | -6.42861 |
| # cluster 44 - Size: 1 |  |  |
|  | XP\_065052612\_6303-model44 | -6.42325 |
|  |  |  |
